# Comparing breath hold versus free breathing irradiation for left-sided breast radiotherapy by PlanIQ™

**DOI:** 10.1186/s13014-023-02386-2

**Published:** 2023-12-14

**Authors:** Ke Yuan, Xinghong Yao, Xiongfei Liao, Pen Diao, Xin Xin, Jiabao Ma, Jie Li, Lucia Clara Orlandini

**Affiliations:** 1 Department of Radiation Oncology, Sichuan Cancer Hospital & Institute, Affiliated Cancer Hospital of University and Electronic Science and Technology of China, Chengdu, China; 2 Sichuan Clinical Research Center for Cancer, Sichuan Cancer Center, Chengdu, China

**Keywords:** Radiotherapy, Breast, Free-breathing, Breath-hold, Feasibility dose-volume histogram

## Abstract

**Background:**

Breast cancer is the most widespread cancer in women and young women worldwide. Moving towards customised radiotherapy, balancing the use of the available technology with the best treatment modality may not be an easy task in the daily routine. This study aims to evaluate the effectiveness of introducing IQ-feasibility into clinical practice to support the decision of free-breathing (FB) versus breath-hold (BH) left-sided breast irradiations, in order to optimise the technology available and the effectiveness of the treatment.

**Methods:**

Thirty-five patients who received 3D radiotherapy treatment of the left breast in deep-inspiration BH were included in this retrospective study. Computed tomography scans in FB and BH were acquired for each patient; targets contoured in both imaging datasets by an experienced radiation oncologist, and organs at risk delineated using automatic segmentation software were exported to PlanIQ™ (Sun Nuclear Corp.) to generate feasibility dose volume histogram (FDVHs). The dosimetric parameter of BH versus FB FDVH, and BH clinical dataset versus BH FDVH were compared.

**Results:**

A total of 30 patients out of 35 patients analysed, presented for the BH treatments a significant reduction (*p* < 0.05) in the heart mean dose (${{\text{D}}}_{{\text{m}}}$), volume receiving 5 Gy (${{\text{V}}}_{5{\text{Gy}}}$) and 20 Gy (${{\text{V}}}_{20{\text{Gy}}}$), of 35.7%, 54.5%, and 2.1%, respectively; for the left lung, a lower reduction was registered and significant only for ${{\text{V}}}_{5{\text{Gy}}}$ (21.4%, *p* = 0.046). For the remaining five patients, the FDVH cut-off points of heart and lung were superimposable with differences of less than 1%. Heart and left lung dosimetric parameters of the BH clinical plans are located in the difficult zone of the FDVH and differ significantly (p < 0.05) from the corresponding parameters of the FDVH curves delimiting this buffer area between the impossible and feasible zones, respectively.

**Conclusion:**

The use of PlanIQTM as a decision-support tool for the FB versus BH treatment delivery modality allows customisation of the treatment technique using the most appropriate technology for each patient enabling accurate management of available technologies.

## Background

Breast cancer is the most widespread cancer in women and young women (< 45 years) worldwide [1, 2]. Adjuvant radiotherapy is part of the standard of care, and the focus on undue doses to organs at risk makes breast treatment one of the most studied because of the important sequelae that can occur even years later. A primary concern is unwanted pulmonary and cardiac irradiation, which may result in late injury [3, 4]. Increased risk of fatal cardiac events, pneumonitis, as well as of a second primary cancer of the breast has been largely reported [5–8]. Considering the incidence of this pathology even at a younger age and the increase in life expectancy [1], it is paramount to limit as much as possible long-term complications reducing pulmonary and cardiac volumes irradiated without compromising the success of the target irradiation. The treatment is delivered in free breathing or deep inspiration breath hold (DIBH) depending on the availability and indications of the centre. The topic of comparing the two techniques regarding their effectiveness, although widely discussed, remains to date very relevant [9, 10].

During DIBH the chest wall expands along with the inferior displacement of the diaphragm, and for left breast treatments, the heart is displaced medially posteriorly and inferiorly away from the target [11]. DIBH allows to minimize the irradiation of nearby organs at risk while maintaining an adequate target dose coverage [12–15] and has therefore become part of clinical practice in many institutions [16–19]. Although it appears clear in the scientific community that there may be an advantage for cardiac dosimetry in performing breath-hold irradiation [20, 21], there are still conflicting opinions and research regarding the advantage of lung dosimetry [22, 23]. The interinstitutional study by Nelms et al. [24] demonstrated that considerable variation in the quality of treatment plans may be attributed to the planner’s general skills. Many studies have focused on a priori estimation of the best possible sparing of organs at risk (OARs) before proceeding to plan optimization, to reduce variability in plan quality [25, 26]. Rocket et al.[27] found that 75% of left-side breast treatments can benefit from breath hold (BH) irradiation suggesting its use as routine clinical practice. On the other hand, some particularly overloaded departments might benefit from a preliminary assessment of the real advantage of proceeding in BH, a practice that nevertheless remains more challenging both in its preparation and execution, and involves the use of specific technology.

Feasibility dose volume histogram (FDVH) is a tool that was introduced in the PlanIQ software (Sun Nuclear Corp., Melbourne, FL) able to estimate for each patient, the lowest possible dose volume histogram (DVH) for OARs, given the full coverage of the target volume with the prescribed dose [28, 29]. This retrospective study aims to evaluate the usefulness of introducing IQ-feasibility into clinical practice to support case by case the decision of free-breathing versus breath-hold left-sided breast irradiation.

## Methods

### Patient selection

This retrospective study included 35 patients with early-stage left breast cancer consecutively admitted to our hospital in 2020. Patients were referred by physicians for whole-breast radiotherapy in BH. The study was approved by the institutional Ethics Committee (approval number: SCCHEC-02-2021-026). Informed consent was obtained from all subjects and/or their legal guardian(s); data were anonymized before use and patient details were de-identified.

### Clinical workflow

The process followed by patients undergoing radiotherapy on the left breast is standardised in our department and has been previously described [30–32]; patient immobilization (supine) was achieved using WingStep (IT-V, Innsbruck, Austria) breast board. Patients were imaged in free breathing (FB) and BH consecutively, with a 16-slice Brilliance Big Bore computed tomography (CT) scanner (Philips Medical Systems, Cleveland, OH) using 3-mm slice thickness; a copper wire was placed along with the palpated breast tissue during the simulation as a support for the target delineation. A surface-guided radiotherapy (SGRT) system was used at simulation CT and throughout the treatment fractions for patient monitoring. CT scans were exported to a commercial platform MIM Version 7.0.5 (MIM Software Inc., Cleveland, Ohio, USA) used for contour segmentation. Target volume and organs at risk were outlined manually on the BH imaging dataset by an experienced radiation oncologist of the breast department following the breast cancer atlas for radiotherapy consensus definitions [33, 34]. Clinical target volume (CTV) included all mammary tissues of the whole breast after lumpectomy as visualized on the CT scans. The planning target volume (PTV) was generated as an isotropic expansion of the CTV with a margin of 3 mm in all directions; the first 5 mm within the outer contour of the body were excluded from both the CTV and the PTV. The heart and left lung contoured on the BH imaging dataset used for the treatment plan clinically delivered will be indicated as ${{\text{Heart}}}_{{\text{man}}}$ and ${{\text{Lung}}}_{{\text{man}}}$, respectively. CT images and the delineated structures were imported into Pinnacle 3™ Version 9.10 (Philips Medical Systems, Eindhoven, the Netherlands) treatment planning system. All plans were created for the Elekta Infinity (Elekta, Stockholm, Sweden) LINAC with a photon beam energy of 6MV and calculated with the full collapsed cone convolution algorithm and a 3-mm dose grid. For all patients the tangential field-in-field technique (TFiF) was used, consisting of two opposing tangential fields and additional fields manually created with the multileaf collimator (MLC) to homogenise the target volume; three to five sub-segments per beam were used, while the tangential field gantry angles ranged between 300° and 315° for the medial beam, and 120° and 135° for the lateral beam. Patients received hypofractionated radiotherapy consisting of a prescription dose (${{\text{D}}}_{{\text{p}}}$) of 42.56 Gy delivered in 16 fractions [35]. The plan was optimised to achieve a mean dose (${{\text{D}}}_{{\text{m}}}$) to the PTV equal to the ${{\text{D}}}_{{\text{p}}}$ with a dose reaching 95% of the target volume (${{\text{D}}}_{95}$) no lower than 95% of the ${{\text{D}}}_{{\text{p}}}$; hotspots were not to exceed 107% of ${{\text{D}}}_{{\text{p}}}$ although they were considered acceptable if the dose received by 2 cm^3^ of the target (${{\text{D}}}_{2{\text{cc}}}$) remained below the 110% isodose line [30].

The dose to the OARs was kept as low as possible without compromising target coverage; constraints normally used in clinical practice were adopted [36] trying to keep the mean dose of the heart under 2 Gy [37], and less than 15% of the left lung receiving more than 20 Gy.

### FB and BH patient’s dataset preparation for FDVH assessment

For each patient, the PTV contoured in the clinical workflow was used to generate the BH FDVH, while for the FB FDVH, the PTV was delineated by the same radiation oncologist who identified it in the corresponding BH imaging. Considering that organ delineation remains operator-dependent, to avoid uncertainties when comparing FB and BH FDVHs, commercial automatic segmentation software (AiPlan, Lianxin Company, Beijing, China) [38] was used to contour the heart (${{\text{Heart}}}_{{\text{auto}}}$) and left lung (${{\text{Lung}}}_{{\text{auto}}}$) on FB and BH CT scans; the contours were successively reviewed and validated by an experienced radiation oncologist. The consistency of automatic segmentation versus manual contouring was assessed on the BH dataset for which manual contours were available for the heart and lungs. Quantitative metrics such as the Dice Similarity Coefficient (DSC), the mean absolute surface-to-surface distance (MASD), and the Hausdorff distance (HD) were used to compare ${{\text{Heart}}}_{{\text{man}}}$ and ${{\text{Heart}}}_{{\text{auto}}}$, and ${{\text{Lung}}}_{{\text{man}}}$ and ${{\text{Lung}}}_{{\text{auto}}}$. DSC provides a measure of overlap between automatic and manual delineations, with 0 indicating no overlap and 1 indicating perfect overlap. MASD and HD are indicative of deviations between the delineations on the surface, with the HD being more sensitive to local surface deviations.

### FB and BH treatment plan quality assessment

**Fig. 1:**
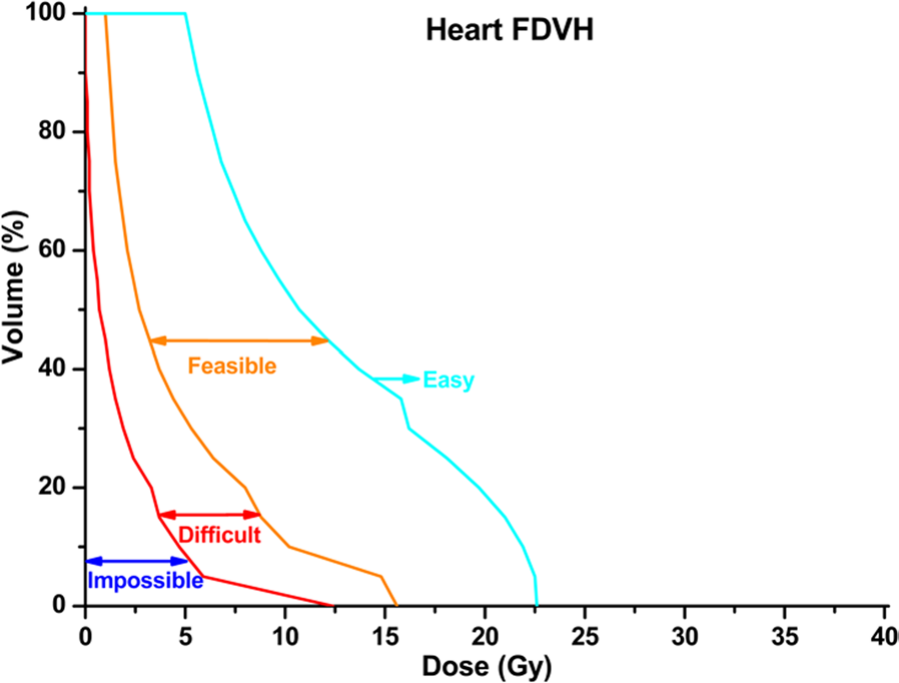
FDVH curves bounding the different zones of dose decay for a representative BH treatment. The “impossible”, “difficult”, “feasible”, and “easy” zones are bounded by the corresponding red, orange, and blue FDVH curves

The FDVH module of PlanIQ™ (Sun Nuclear Corp., Melbourne, FL) is marketed as software for the analysis of treatment plan quality metrics. FDVH is based on a falloff of the ideal dose from the prescribed dose at the target boundary, allowing the quantitative determination of the best possible OAR FDVH that can be generated based on the benchmark dose, and making FDVH curves more easily achievable. FDVH can simulate the dose prescribed to a clinical site in four zones of dose decay modes, starting from the boundary of the target volume. Based on the geometric relationship between the organs at risk and the target volume, the exposure of the organ at risk and the corresponding dose received can be quantified in each zone [26]. The four zones are defined as “impossible”, “difficult”, “feasible”, and “easy” and for clear identification, they are bounded by red, orange, and blue FDVH curves, respectively, as depicted in Fig. 1 for a representative patient treated in BH.

In this study, the FDVH module was used to compare the dosimetric difference resulting from a FB versus BH treatment plan approach of left-sided breast cancer patients. For each patient, FB and BH CT datasets and patient structures were exported to PlanIQ™ to generate the corresponding OAR FDVHs; heart and left lung ${{\text{D}}}_{{\text{m}}}$, ${{\text{V}}}_{5{\text{Gy}}}$ and ${{\text{V}}}_{20{\text{Gy}}}$ (percentage volume receiving 5 and 20 Gy, respectively) were evaluated assuming the entire PTV was receiving 40.43 Gy equal to 95% of the ${{\text{D}}}_{{\text{p}}}$. BH and FB OARs dosimetry was compared for the red and orange FDVH curves bounding the impossible and difficult zone, respectively, deemed more challenging by PlanIQ™. Statistical analysis of the data was performed by using SPSS software (IBM, Armont, USA) version 19.0, used to test the results of the radiation treatment plans of the BH and FB groups by paired t-test; a difference of *p* < 0.05 was considered statistically significant.

## Results

### Accuracy of the datasets used

Automatic segmentation of the whole heart and left lung volumes are consistent with contours performed manually in the clinical practice, with minor deviations not statistically significant. In Table 1, the results of DSC, MASD, HD and *p*-value are reported. Results are given as the mean ± standard deviation.

**Table 1:** Consistency of heart and left lung, manual versus automatic contouring for BH dataset

	DSC^a^	MASD^b^ (mm)	HD^c^ (mm)
Heart	0.965 ± 0.006	2.573 ± 0.020	2.947 ± 0.272
Left Lung	0.977 ± 0.004	1.693 ± 0.100	1.327 ± 0.191

^a^DSC, dice similarity coefficient; ^b^MASD, mean absolute surface-to-surface distance; ^c^HD, Hausdorff distance

### BH Clinical DVH versus BH FDVH

The results dosimetric parameters of the heart and left lung of the clinically delivered plans and the corresponding dosimetric parameters evaluated by the red and orange FDVH curves at the boundary between the impossible and feasible areas are shown in Table 2.

**Table 2:** Comparison of the heart and left lung dosimetric parameters for the clinical BH DVH and BH FDVH red and orange curves

		^d^BH DVH	BH ^e^FDVH			
		Clinical	Orange		Red	
		Mean ± SD	Mean ± SD	*p*	Mean ± SD	*p*
^a^ ${\mathbf{V}}_{5\mathbf{G}\mathbf{y}}$** (%)**	Heart	8.2 ± 4.2	29.9 ± 4.4	0.016	7.2 ± 5.1	< 0.001
	Left Lung	26.9 ± 5.1	35.5 ± 4.7	< 0.001	21.3 ± 5.8	< 0.001
^a^ ${\mathbf{V}}_{20\mathbf{G}\mathbf{y}}$** (%)**	Heart	1.6 ± 1.8	1.7 ± 1.0	< 0.001	0.8 ± 1.0	< 0.001
	Left Lung	11.3 ± 3.9	12.3 ± 1.4	< 0.001	3.8 ± 0.7	< 0.001
^b^ ${\mathbf{D}}_{\mathbf{m}}$** (Gy)**	Heart	2.2 ± 1.5	4.4 ± 0.7	< 0.001	1.5 ± 0.7	< 0.001
	Left Lung	6.3 ± 0.9	6.2 ± 1.6	< 0.001	3.4 ± 0.7	< 0.001

A *p*-value < 0.05 is considered statistically significant. The average value and standard deviation are reported for each group dataset

^a^ ${{\text{V}}}_{{\text{xGy}}}$, volume in percentage receiving x Gy; ^b^ ${{\text{D}}}_{{\text{m}}}$, mean dose; ^d^BH, breath hold; ^e^FDVH, feasibility dose volume histogram

Clinical treatment plans in BH meet the target dose coverage and dose sparing requirements for OARs; the dosimetric parameters of the heart and left lung of the clinical plans are in the FDVH difficult zone, while they differ significantly from the corresponding parameters of the red and orange FDVH curves (*p* < 0.05), respectively, which delimit this area, a buffer between the impossible and feasible zones.

### Comparison of BH and FB contours datasets

In Table 3, PTV and OAR volumes corresponding to the FB and BH are reported. In BH, PTV and heart median volumes did not present a significant difference (*p* = 0.064 and *p* = 0.106, respectively); the volume of the left lung increased significantly in BH (*p* = 0.001) with a median value of 75.1%, range (25.59—139.70) %.

**Table 3:** PTV and OAR volumes evaluated from the free-breathing and breath-hold CT datasets and corresponding *p*-value

	Free breathing volume (cc)	Breath hold volume (cc)	Breath hold versus free breathing ΔVOLUME (%)	*p*
^a^PTV	524 (203.4 ÷ 990.1)	535.7 (252.4 ÷ 1005.1)	− 0.03 (− 6.08 ÷ 8.25)	0.064
Heart	589.4 (414.1 ÷ 866.6)	584.6 (410.9 ÷ 917.9)	0.14 (− 8.30 ÷ 7.91)	0.106
Left Lung	1046.4 (600.1 ÷ 1837.5)	1835.3 (1033.4 ÷ 2629.0)	75.10 (25.59 ÷ 139.70)	0.001

^a^PTV, planning target volume

### BH FDVH versus FB FDVH

**Fig. 2:**
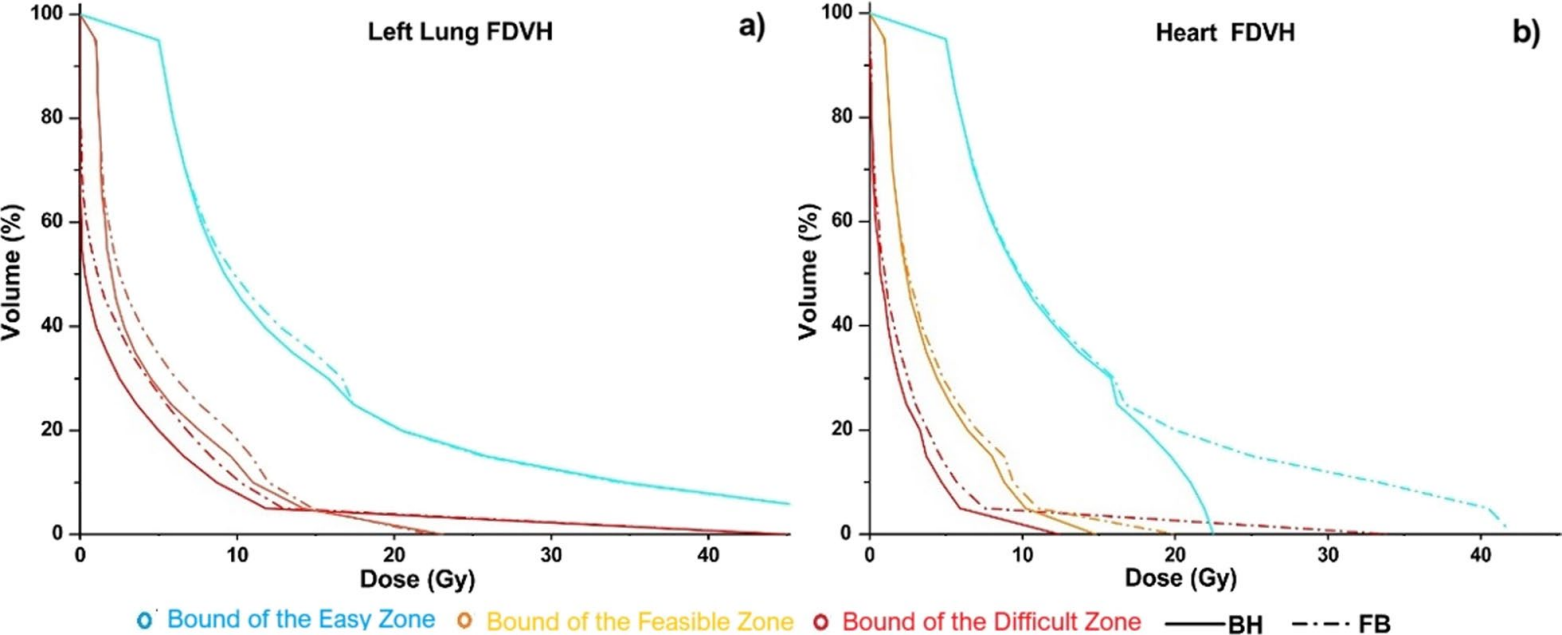
Left lung (**a**) and heart (**b**) FB and BH FDVH curves bounding the different dose decay mode zones for a representative patient

The target dose distribution of FB and BH FDVHs meets clinical requirements and does not present a statistical difference (p > 0.05) in the target ${{\text{D}}}_{95}$, ${{\text{D}}}_{2{\text{cc}}}$. For 30 patients out of 35 patients the heart and left lung BH FDVHs dosimetric parameters, are reduced compared with the corresponding FB FDVHs; the results obtained are shown in Tables 4 and 5 for the red and orange FDVHs curves, respectively. The largest differences in the FDVH were found for heart ${{\text{V}}}_{5{\text{Gy}}}$ and ${{\text{D}}}_{{\text{m}}}$ with mean percentage reductions of 54.5% and 35.7%, respectively, in the red curves, and 16.7%, and 16.0%, respectively, in the orange curve; similarly, for the left lung ${{\text{V}}}_{5{\text{Gy}}}$ and ${{\text{D}}}_{{\text{m}}}$ a mean percentage reduction of 21.4% and 15.1%, respectively, for the red curve, and 13.7%, and 7.1%, respectively, for the orange FDVH. Left lung ${{\text{D}}}_{{\text{m}}}$ and ${{\text{V}}}_{20{\text{Gy}}}$ did not present significant differences in the boundaries for the red and orange boundary curves. The comparison of FB and BH heart and left lung FDVH curves obtained for a representative patient are shown in Fig. 2.

**Table 4:** Comparison of the heart (a) and left lung (b) FB versus BH Feasibility Dose Volume Histogram (FDVH) red curves

			FDVH red			
			BH Mean ± SD	FB Mean ± SD	FB versus BH % diff Mean (range)	*p*-value
^a^ ${{\text{V}}}_{5{\text{Gy}}}$ (%)		Heart	7.2 ± 5.1	15.1 ± 6.5	− 54.5 (− 25.0; − 50.0)	0.013
		Left Lung	21.3 ± 5.8	25.8 ± 5.4	− 21.4 (0.0; − 46.4)	0.046
^a^ ${{\text{V}}}_{20{\text{Gy}}}$ (%)		Heart	0.8 ± 1.0	1.0 ± 1.5	− 2.1 (− 1.0; − 4.0)	0.033
		Left Lung	3.8 ± 0.7	4.0 ± 1.2	− 10.0 (− 5.0; − 22.0)	0.068
^b^ ${{\text{D}}}_{{\text{m}}}$ (Gy)		Heart	1.5 ± 0.7	2.5 ± 0.8	− 35.7 (− 19.4; − 73.3)	0.028
		Left Lung	3.4 ± 0.7	3.9 ± 0.7	− 15.1 (− 4.0; − 23.3)	0.076

^a^ ${{\text{V}}}_{{\text{xGy}}}$, volume in percentage receiving x Gy; ^b^ ${{\text{D}}}_{{\text{m}}}$, mean dose; ^c^FB, free breathing; ^d^BH, breath-hold

**Table 5:** Comparison of the heart (a) and left lung (b) FB versus BH Feasibility Dose Volume Histogram (FDVH) orange curves

			FDVH orange			
			BH Mean ± SD	FB Mean ± SD	FB versus BH % diff Mean (range)	*p*-value
^a^ ${{\text{V}}}_{5{\text{Gy}}}$ (%)		Heart	29.9 ± 4.4	35.8 ± 4.8	− 16.7 (− 7.9; − 30.6)	0.003
		Left Lung	35.5 ± 4.7	39.8 ± 3.5	− 13.7 (− 0.5; − 36.4)	0.044
^a^ ${{\text{V}}}_{20{\text{Gy}}}$ (%)		Heart	1.7 ± 1.0	1.8 ± 2.4	− 1.7 (− 0.2; − 5.5)	0.024
		Left Lung	12.3 ± 1.4	16.2 ± 1.7	− 22.2 (− 20.0; − 30.0)	0.068
^b^ ${{\text{D}}}_{{\text{m}}}$ (Gy)		Heart	4.4 ± 0.7	5.3 ± 0.8	− 16.0 (− 7.8; − 36.2)	0.042
		Left Lung	6.2 ± 1.6	6.6 ± 0.6	− 7.1 (− 14.5; − 19.7)	0.087

^a^ ${{\text{V}}}_{{\text{xGy}}}$, volume in percentage receiving x Gy; ^b^ ${{\text{D}}}_{{\text{m}}}$, mean dose; ^c^FB, free breathing; ^d^BH, breath-hold

### Timing of the procedure

The time required to automatically segment the heart and left lung is about 5 min, mainly due to the time needed to import the CT scan into the automatic segmentation software and the subsequent export of CT images and structures to PlanIQ™. The average time needed for the radiation oncologist to verify/adjust the target on the FB CT dataset is around 10 min. The time to generate the FDVH is 2 min for each structure. The total average time of the procedure setting to compare the FB and BH FDVH for the heart and left lung is 20 min with a range of 18 to 22 min.

## Discussion

Radiotherapy is rapidly evolving towards precise targeting, precise planning and precise treatment [39, 40]. Accurate treatment depends on the dose distribution received by the tumor tissue, which is determined by many factors including the delivery technique chosen. In recent years, automatic planning optimization solutions have received much attention to address the high cost and low efficiency of the reverse planning process [18–20].

FDVH is a tool to estimate the best possible sparing dose of OARs a priori before starting the plan optimization [41]. The algorithm does not require a database of prior plans but rather derives the FDVH from nearly first principles, assuming that the targets are uniformly covered with the prescription doses. It is easily parametrized based on a short list of model geometrical datasets. The method is agnostic to the planning technique and beam arrangement, requiring only the regions of interest, the energy, and optionally the CT dataset as inputs. 

In this study, FDVH was used as an independent method to compare in large clinical left-sided breast datasets the dosimetry of the heart and left lung for FB and BH treatments. The dosimetric parameters provided by the FDVH in the FB datasets and the BH datasets were compared and analysed to explore and quantify differences in the OARs dosimetry. The time needed for the whole procedure is about 20 min per patient. The results obtained showed that the dose distribution of the FB and BH FDVH met the clinical prescription requirements, and there was no statistical difference in the target area dosimetric parameters. OARs dose can be reduced with BH datasets, particularly heart ${{\text{V}}}_{5{\text{Gy}}}$ and ${{\text{D}}}_{{\text{m}}}$. The results obtained are not systematically valid for all patients; a percentage of patients, which in the case of our study stands at 14.2% have no advantage in following the breath-hold irradiation procedure. The method is well-suited for approximating the best-possible OAR DVH curve; however, because it enforces 100% of the target coverage whereas a real-world plan often sacrifices target coverage near OARS there can be deviations of the clinical DVH versus FDVH. In particular, the geometry of the OAR in relation to the target is the major driver of the achievable OAR FDVH [42, 43]. Its simplicity is nevertheless a cause of the algorithm’s limitation; because it is designed to minimize the OARs DVH as much as possible, based on the geometry and distance between the OAR and the target volume.

Tools capable of providing predictions of what is dosimetrically achievable (and ideally optimal) are greatly needed in radiation treatment planning not only to reduce plan variability and ensure quality but also as a tool to support the radiation oncologist in the decision process. The use of artificial intelligence (AI) in setting up predictive models for BH treatment decisions is to date an investigated option [44]. Vendrame et al. [45], after generating FB and BH treatment plans, considered the decrease of the maximum dose to the left anterior descending artery as a parameter to select patients eligible for BH or not; the predictive model set up used AI to analyse different phases of the respiratory cycle.

This work showed with an independent method that left-sided-breast treatments performed in BH and FB enable target dose distribution that met the clinical requirements without statistical differences; the dosimetric advantages that can arise in the heart and left lung dosimetry for BH delivery exist but not indiscriminately for all cases; for even a small percentage of patients, the use of the breath-hold technique does not lead to additional benefits. A personalised assessment is important to decide on the appropriate type of treatment and to optimise the use of the available technology, avoiding more demanding treatments for cases that will not have any benefits.

## Conclusion

The use of PlanIQTM as a decision-support tool for the FB versus BH treatment modality may allow the customisation of the treatment technique using the most appropriate delivery techniques for each patient while enabling an accurate use of available technologies.

## Data Availability

The data are fully available and will be included within the article and in its additional files.

## Abbreviations

BH: Breath-hold
OARs: Organs at risk
FDVH: Feasibility dose volume histogram
DVH: Dose-volume histogram
CTV: Clinical target volume
CT: Computed tomography
PTV: Planning target volume
$${{\text{D}}}_{{\text{p}}}$$: Prescription dose
$${{\text{D}}}_{{\text{m}}}$$: Mean dose
FB: Free breathing
${{\text{Lung}}}_{{\text{auto}}}$: Automatic Lung contour
${{\text{Heart}}}_{{\text{auto}}}$: Automatic heart contour
${{\text{Lung}}}_{{\text{man}}}$: Manual contour of the Lung
${{\text{Heart}}}_{{\text{man}}}$: Manual contour of the heart
MASD: Mean absolute surface-to-surface distance
DSC: Dice similarity coefficient
HD: Hausdorff distance
